# Bloch surface wave structures for high sensitivity detection and compact waveguiding

**DOI:** 10.1080/14686996.2016.1202082

**Published:** 2016-07-29

**Authors:** Muhammad Umar Khan, Brian Corbett

**Affiliations:** ^a^ Tyndall National Institute, University College Cork, Cork, Ireland

**Keywords:** Bloch surface waves, Optical sensors, silicon photonics, Resonant sensor, Surface sensing, Waveguide sensor, Dielectric multilayer, Planar optical circuit, Label free sensing, 40 Optical, magnetic and electronic device materials, 201 Electronics / Semiconductor / TCOs, 204 Optics / Optical applications, 208 Sensors and actuators, 212 Surface and interfaces, 505 Optical / Molecular spectroscopy

## Abstract

Resonant propagating waves created on the surface of a dielectric multilayer stack, called Bloch surface waves (BSW), can be designed for high sensitivity monitoring of the adjacent refractive index as an alternative platform to the metal-based surface plasmon resonance (SPR) sensing. The resonant wavelength and polarization can be designed by engineering of the dielectric layers unlike the fixed resonance of SPR, while the wide bandwidth low loss of dielectrics permits sharper resonances, longer propagation lengths and thus their use in waveguiding devices. The transparency of the dielectrics allows the excitation and monitoring of surface-bound fluorescent molecules. We review the recent developments in this technology. We show the advantages that can be obtained by using high index contrast layered structures. Operating at 1550 nm wavelengths will allow the BSW sensors to be implemented in the silicon photonics platform where active waveguiding can be used in the realization of compact planar integrated circuits for multi-parameter sensing.

## Graphical abstract

**Figure:**
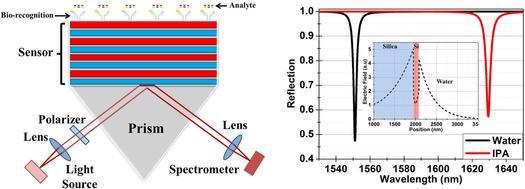

## 1. Introduction

There has been ongoing research activity aimed at the realization of optical sensors for the measurement of chemical and biological analytes for many decades. The first optical chemical sensor was reported in 1975 for the measurement of CO_2_ and O_2_ concentration using the changes in absorption spectrum.[1] Since then, various optical approaches including ellipsometry, spectroscopy, interferometry and surface electromagnetic waves (SEW) have been used.[2] SEW propagate along the interface between two media and can be strongly confined with a significantly enhanced field at the surface which decays exponentially into the neighbouring media. The optical properties of the two media determine the nature of the SEW that can be sustained, resulting in unique properties that enable high surface sensitivity, real-time and label-free detection. The most popular surface wave based sensing technology is the surface plasmon resonance (SPR) method [2–4] which is based on the excitation of the surface plasmon polaritons along a metal/dielectric interface by incident light with a wave-vector matching that of the SPR. The use of SPR for gas sensing and bio-sensing was demonstrated in 1982.[5–7] Since then, SPR sensing has been receiving continuously growing attention from the scientific community. Noise estimations reveal that best performing plasmon based systems have nearly reached their theoretical limits.[8] One route to better performing SPR sensors could be to laterally pattern the structure [9,10] in order to exploit local field enhancement. Alternatively, Bloch surface waves (BSW) guided at the surface of a dielectric one-dimensional photonic crystal [11] have been suggested for plasmon like sensing [12]. These modes exploit the band gap of a photonic crystal to obtain guiding of a surface wave thus giving rise to a surface sensitive evanescent field.

BSW were first theoretically reported [13] and followed by experimental demonstration in 1978.[14] Twelve pairs of alternating layers of 500 nm thick GaAs and 500 nm thick Al_0.2_Ga_0.5_As on a GaAs substrate were used to generate the BSW at a wavelength of 1150 nm. Two-dimensional (2D) photonic crystal structures also sustain BSW.[15,16] There are several advantages provided by BSW propagation on flat dielectric surfaces. First, due to the use of dielectric materials the losses are very low, allowing for the propagation of BSW over long distances. The use of a Bragg reflector provides greater freedom in the operating wavelengths than SPR which is limited to a narrow range of metal dependent wavelengths. The Bragg reflector can be designed to sustain both TE and TM polarized BSW, a condition that cannot be achieved with SPR as they are only TM polarized. Because the maximum intensity associated with the BSW can be engineered to be at the surface, it is particularly attractive for biosensing using the large field enhancements.[17,18] A related concept is the resonant mirror [19,20] introduced in 1993. It uses a high index layer on a low index layer for excitation of the resonance through the substrate.

In this paper, we discuss the design of BSW structures and their use for sensing and waveguiding applications. By presenting already reported structures, we show that the BSW can be designed for both TE and TM polarized light. The role of the refractive index contrast and number of layers is explained through simulations. Design of a sensing platform using only a single pair of high index contrast (Si/SiO_2_) layers is explained. We show using simulations that this high index contrast sensor principle can be applied to silicon-on-insulator (SOI) on silicon substrates at a wavelength of 1550 nm where Si is transparent and sharper resonances with better sensitivities (1950 nm/RIU) can be obtained. We introduce a new waveguide coupling of a two-layer BSW-like structure for on-chip applications. The application of BSW to sensing is discussed where different measurement techniques can be employed. Finally, we discuss the use of BSW for planar optical circuits.

## 2. Design of Bloch surface wave sensors

A BSW can be excited on the surface of a highly reflective one-dimensional photonic band-gap or Bragg reflector designed with the optical thickness of each layer being one quarter of the incident wavelength, λ. A transmitting mode inside the band-gap can be initiated by breaking the periodicity of the Bragg reflector and can be made to travel on the surface of the Bragg reflector by truncating the reflector at the appropriate thickness. The mode has an evanescent tail into the upper medium as in the case of SPR. The mode is resonant based on the thickness of the layers. A conventional waveguide mode also has an evanescent tail but it is non-resonant and additional lateral structuring is required to make such a structure resonant.

**Figure 1.**
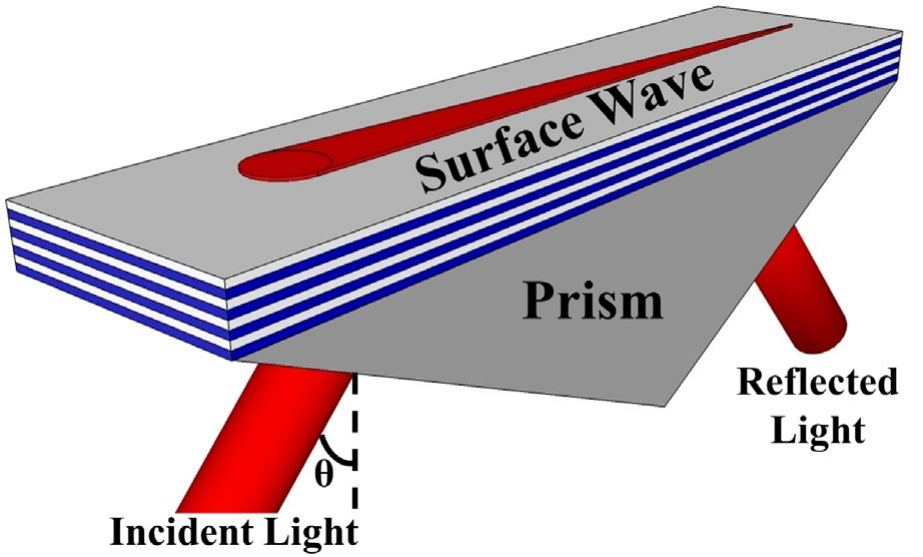
Schematic showing the excitation of the Bloch surface wave (BSW) using the Kretschmann–Raether (K-R) configuration. The surface wave is excited for an incident light having a wave vector equal to that of the surface wave. This excitation is detected by a dip in the intensity of reflected light at the resonant wavelength or by a change in intensity of a single wavelength as the angle is rotated in a θ – 2θ manner through the resonance.

BSW have been demonstrated using 2D,[15,16] 3D [21] and more widely 1D photonic crystal structures. BSW have been investigated with TiO_2_/SiO_2_,[22–25] Ta_2_O_5_/SiO_2_,[26–29] Si_3_N_4_/SiO_2_[30–33] and porous Si [34–38] multilayers for refractive index sensing, fluorescence sensing [26,32,39] and waveguiding applications.[40–42] The BSW are mostly excited through the substrate using the prism based Kretschmann–Raether (K-R) excitation configuration [43,44] shown in Figure 1 which allows unimpeded access to the surface and measurement of the angular or wavelength dependence of the resonance for different surface chemistries. We first discuss the design of a couple of TiO_2_/SiO_2_ BSW sensors reported in the literature. These sensors operate for different polarizations of light so it will help understanding the design of BSW sensors for both orthogonal polarizations. We then explain the design of a high index contrast BSW-like sensor.

**Figure 2.**
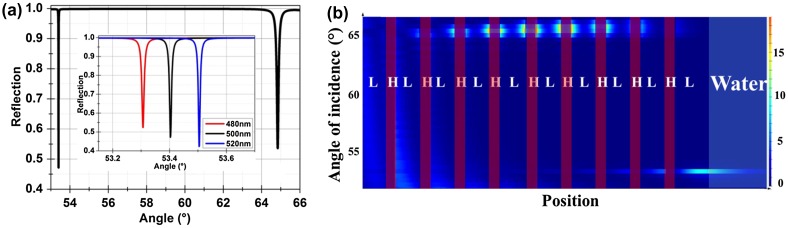
(a) Calculated reflection response for a structure having 10 pairs of TiO_2_ / SiO_2_ layers under plane wave excitation. Two resonances representing surface and sub-surface resonances are obtained at angles of incidences of 53.4° and 65° respectively. The angles are quoted with respect to the surface normal in the substrate. The inset shows the shift in the surface resonance for different thicknesses of the last SiO_2_ layer. (b) Calculated field distributions at resonance (980 nm). High (TiO_2_) and low (SiO_2_) index layers are marked by H and L respectively. The field distribution shows that the resonance at 65° is due to a sub-surface mode with the electric field buried inside the dielectric stack. This sub-surface mode is similar to a guided waveguide mode with a Gaussian distribution but is modulated by the periodicity of the stack. The electric field distribution of such sub-surface mode is presented in [38]. The resonance at 53.4° is due to a surface wave with the electric field penetrating into the medium above the surface.

A sensor consisting of 20 layers of TiO_2_ (*n*_H_ = 2.30) and SiO_2_ (*n*_L_ = 1.434) with respective thicknesses of *d*_H_ = 163 nm and *d*_L_ = 391 nm sustains a TM-polarized BSW at the wavelength of 980 nm.[24] The final layer adjacent to the external medium is 500 nm thick SiO_2_. The calculated reflection response for an incident wavelength of 980 nm is shown in Figure 2(a) where water (*n* = 1.33) was used as the medium above the surface. Two resonance dips at incident angles of 53.4° and 65° can be observed in the reflection response having a full width at half maximum (FWHM) of 0.009° and 0.11° respectively. The calculated field distributions for these modes are shown in Figure 2(b). It can be noticed that the electric field for the resonance at 53.4° is concentrated at the surface of the dielectric stack. The calculated effective refractive index (*n*_eff_ = n_prism_sin(θ)) of the mode is 1.34 with a 1 μm penetration depth of the electric field (at 1/e) into the water. A significant part of the electric field for surface mode penetrates into the water, so a change in refractive index above the surface will result in shifting of the surface resonance. The electric field for the resonance at 65° on the other hand is buried inside the stack, so this mode is named as a Bloch sub-surface mode (BSSW). As the field is buried inside the stack so a change in refractive index above the surface will not affect the sub-surface wave. The effective index of the sub-surface wave was calculated to be 1.52, larger than surface wave as field is residing inside the stack instead of comparatively lower index water. We have changed the thickness of the last SiO_2_ layer to show the effect of this thickness on the resonances. The calculated resonances for a 480, 500 and 520 nm thick last SiO_2_ layer are shown in the inset of Figure 2(a). The sub-surface resonance is not affected by this variation while the surface resonance moves to larger angles with an increase in the thickness. This shows that the position of the surface wave resonance can be tuned inside the band-gap by changing the last layer thickness.

**Figure 3.**
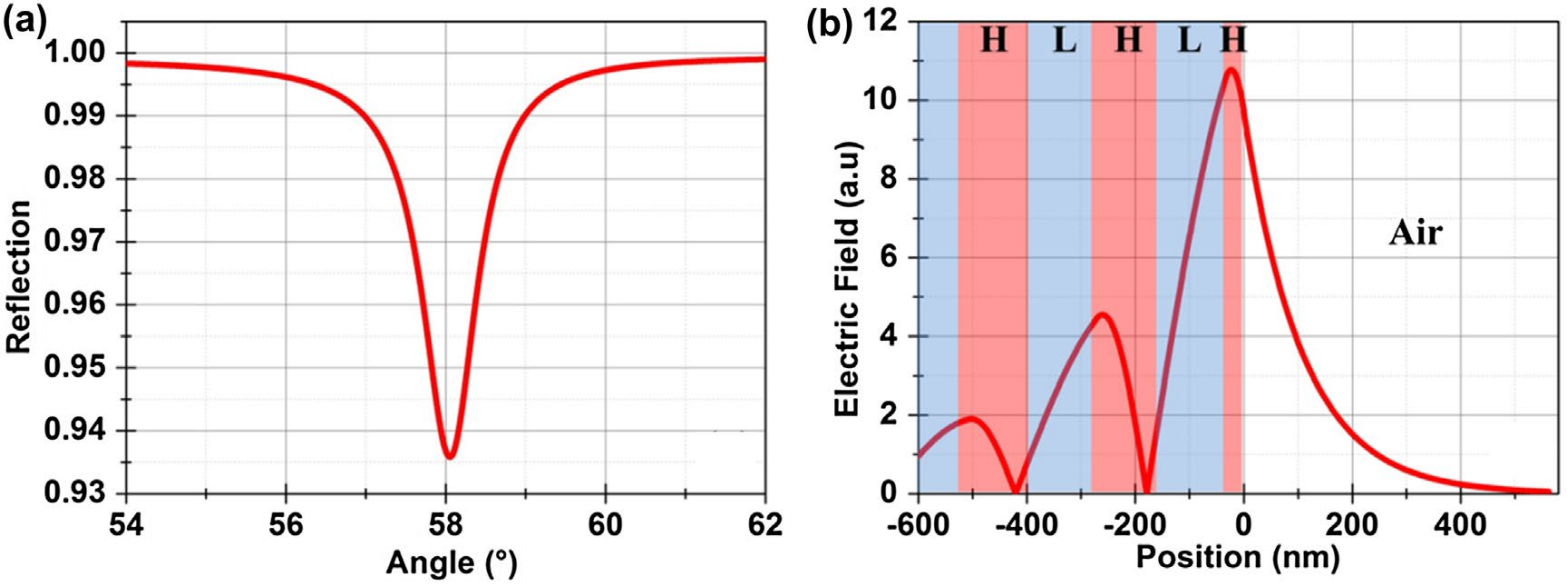
(a) Calculated reflection response for a structure having five layers of TiO_2_ and SiO_2_ layers for operation at 550 nm wavelengths. Air was assumed above the surface for these calculations. The angles are quoted with respect to the surface normal in the BK7 substrate. (b) Calculated field distribution at resonance. High and low index layers are marked by H and L respectively.

A five-layer sensor using TiO_2_ and SiO_2_ with respective thicknesses of *d*_H_ = 96 nm and *d*_L_ = 140 nm was reported to sustain a TE-polarized BSW at the wavelength of 550 nm.[25] The surface of the multilayer adjacent to the external medium was a 34.6 nm thick TiO_2_ layer. The BSW in this design is sustained for TE polarized light with the number of layers reduced from 20 to five using the same materials. The last layer of the sensor is a high index layer in contrast to the low index layer for the previously discussed BSW. The calculated reflection response for this structure is shown in Figure 3(a). A shallow TE polarized resonance is calculated at an incident angle of 58° with air (*n* = 1) above the surface. The FWHM of the resonance is calculated to be 0.78° which is larger than the earlier presented structure. The effective refractive index of the mode is only 1.29 as a significant portion of the field is residing inside the air. The electric field distribution at resonance is shown in Figure 3(b). The penetration depths of the electric field (at 1/e) are calculated to be 100 and 300 nm into air and water respectively. The penetration depth of this BSW into water is smaller than the previous one as the field is more tightly confined to the surface due to the high index terminating layer. Thus, the use of a high index surface layer helps to strongly confine the field to the surface.

Learning from the above discussed structures, we reduced the number of layers to two using high (ZrO_2_) and low (SiO_2_) index dielectric layers on a sapphire substrate with respective refractive index values of 2.02+0.02i, 1.47 and 1.77 at *λ* = 820 nm. The imaginary part in the refractive index represents the loss associated with ZrO_2_ at 820 nm. Thicknesses of 170 nm and 676 nm were calculated to obtain a TM-polarized BSW-like resonance using an aqueous environment (*n* = 1.33). The designed thicknesses of ZrO_2_ and SiO_2_ were deposited on sapphire substrate and the resonance was experimentally observed. Although the observed resonance was weak due to low number of layers, the resonance can be made sharper and deeper either by increasing the number of layers or by increasing the refractive index contrast for same number of mirrors.

**Figure 4.**
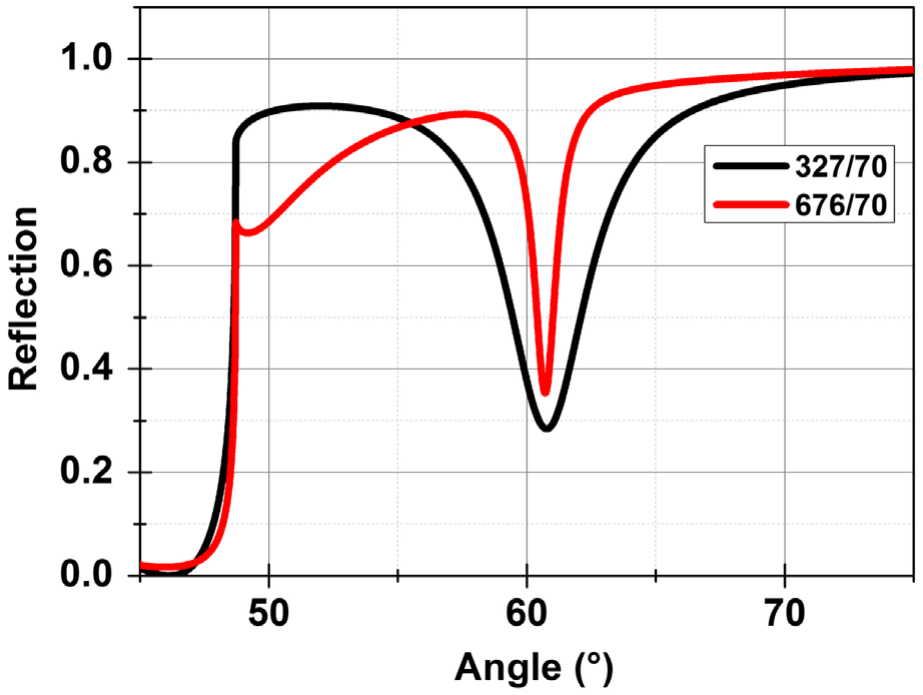
Comparison of the reflection response for a two-layer Si/SiO_2_ BSW for 327 nm and 676 nm thick SiO_2_ layers. A 70 nm thick Si layer was used for these calculations.

A larger index contrast between the dielectric layers can result in sharper resonances for this two-layer approach which can be achieved using Si and SiO_2_ as the high and low index layers. A structure was designed using a Si layer on SiO_2_ on sapphire substrate having refractive index values of 3.67+0.03i, 1.47 and 1.77 respectively for a wavelength of 820 nm. The imaginary part in the refractive index represents the significant loss associated with Si at 820 nm. The thicknesses for the Si and SiO_2_ layers were calculated to be 70 nm and 327 nm for an aqueous environment (*n* = 1.33). The calculations show a TM polarized surface mode at an incident angle of 61° for *λ* = 820 nm with a FWHM of 3.5°. The FWHM can be reduced by designing the structure for the second order mode by increasing the thickness of the SiO_2_ layer to 676 nm resulting in a FWHM of 1° (Figure 4). It should be noticed that the FWHM is still high due to the loss associated with silicon at visible wavelengths. We will show later that sharper resonances can be achieved by operation around telecom wavelengths where silicon is transparent.

**Figure 5.**
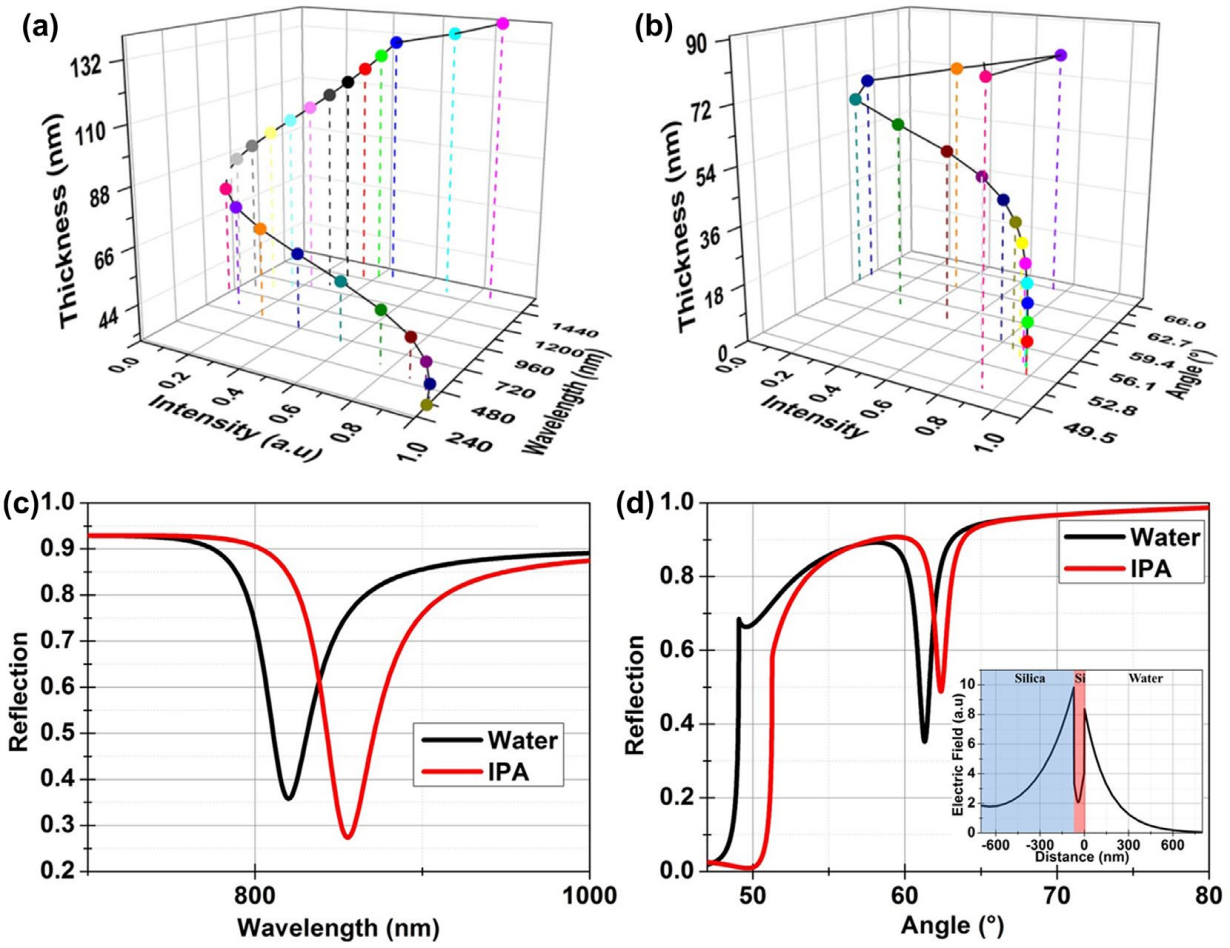
(a) 3D graph shows that the resonance depth is maximum (intensity minimum) for a Si layer thickness of 70 nm on 676 nm SiO_2_ for operation at 820 nm. The angle of incidence for the calculations was fixed at 61°. (b) 3D graph shows that strongest resonance is observed for Si thickness of 70 nm at a resonance angle of 61°. (c) Calculated wavelength dependent reflection for the designed sensor for an angle of incidence of 61° in both water (*n* = 1.33) and IPA (*n* = 1.37) environments. (d) Calculated angle dependent reflection response for 820 nm incident light. Water (*n* = 1.33) and IPA (*n* = 1.37) were used to calculate the expected shift in the resonance for a change of Δ*n* = 0.04 above the surface. The inset shows the calculated field distribution for the BSW at resonance.

The effect of the silicon layer thickness on the resonance wavelength was studied by varying the silicon layer thickness from 40 to 140 nm. The angle of incidence and thickness of SiO_2_ were fixed at 61° and 676 nm respectively. The 3D plot in Figure 5(a) shows that the reflection intensity at resonance is at a minimum for a 70 nm thick silicon layer at 820 nm supporting the earlier calculations. The effect of the silicon layer thickness on the resonance angle was also studied with the wavelength of incident light fixed at 820 nm. The 3D plot in Figure 5(b) confirms that the strongest resonance will be observed for 70 nm thick silicon layer at a resonance angle of 61°. So, 676 nm and 70 nm thick layers of SiO_2_ and Si were used to calculate the wavelength and angle dependent reflections shown in Figure 5(c) and (d). The shift in the resonance angle and wavelength for a change in the refractive index of Δ*n* = 0.04 in the overlying medium, as appropriate to isopropyl alcohol (IPA), was calculated to be Δ*θ* = 1.09° for *λ* = 820 nm and a shift of Δ*λ* = 36 nm for *θ* = 61°. Thus, the calculated bulk sensitivities are 900 nm/RIU (refractive index unit) and 27.3°/RIU.

The field distribution for the excited mode is shown in the inset of Figure 5(d). The field distribution shows that the mode is evanescent with only a small portion of the power residing inside the high index silicon layer, in contrast to the TE mode explained earlier. We remind the reader that a simple Si waveguide is broadband while our layered structure is resonant. The penetration depth into water was calculated to be 150 nm with an effective refractive index of the mode being 1.54. This strong confinement of the field to the surface makes this structure very suitable for label-free detection of biological analytes. The calculated electric field penetration for this high index contrast BSW is smaller than the TiO_2_/SiO_2_ BSW discussed above because of the high index (Si) top layer.

**Figure 6.**
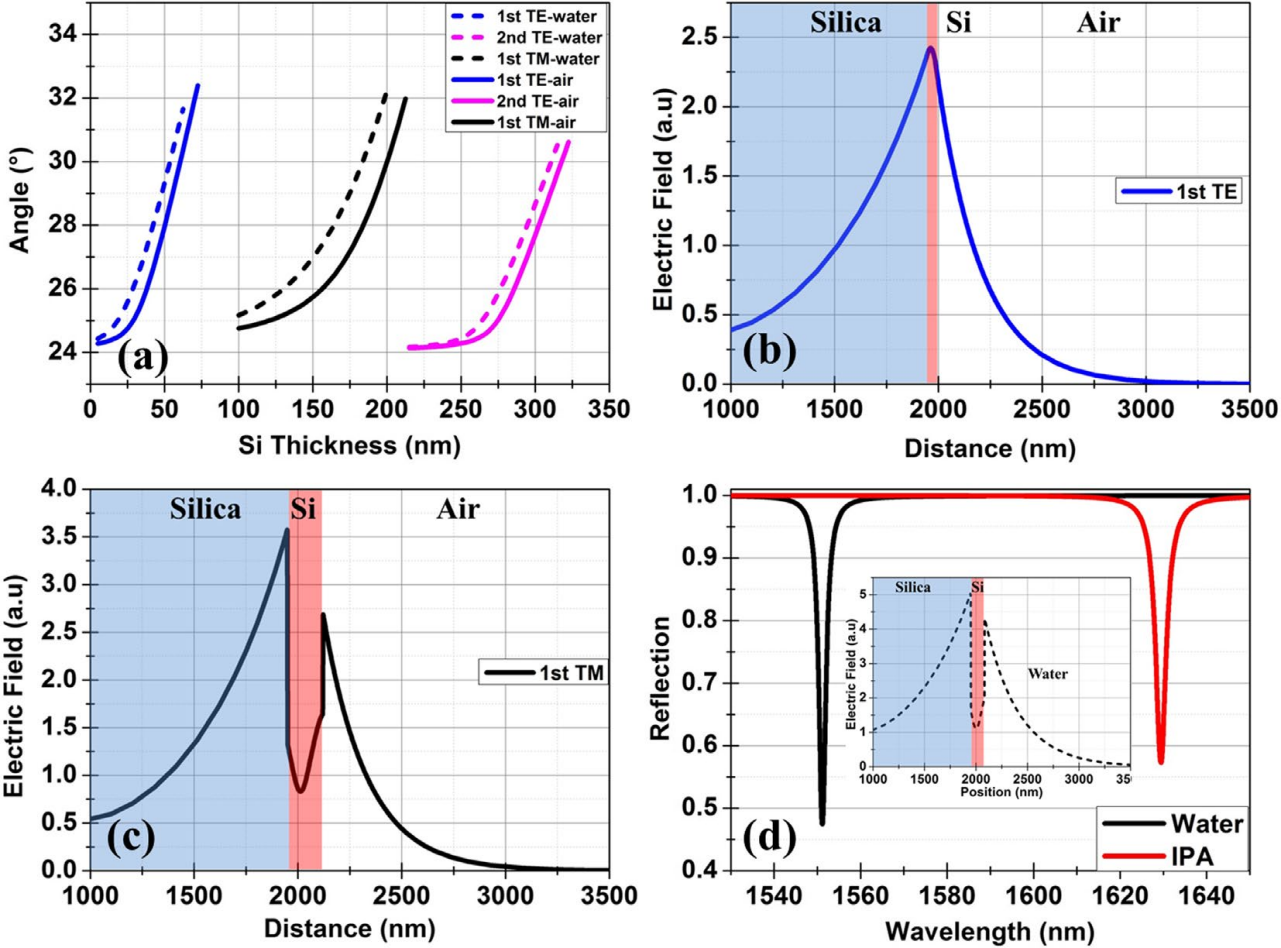
(a) Calculated BSW enhanced TE and TM modes for a wavelength of 1550 nm on SOI. The thickness of the SiO_2_ layer is 2000 nm. The angles are quoted with respect to the surface normal in Si substrate. (b) Electric field of the fundamental TE mode guided by a 40 nm thick silicon layer at an angle of 26° (c) Electric field of fundamental TM mode calculated for 170 nm thick silicon layer at an angle of 26.35°. (d) Calculated wavelength dependent reflection in both water (*n* = 1.33) and IPA (*n* = 1.37) environments. The inset shows the calculated electric field of fundamental TM mode using 133 nm thick silicon layer at an angle of 26.1°. Water was used as the overlying medium for this calculation.

The single pair high index contrast structure can be designed to be resonant at a desired wavelength and polarization state. We designed the structure for operation λ around 1550 nm where silicon is transparent and the silicon on insulator (SOI) platform is widely used for photonic integrated circuits. We simulated a conventional (2 μm thick SiO_2_) SOI arrangement with a silicon substrate rather than sapphire as silicon is transparent for these wavelengths, allowing access through the substrate. Refractive index values of 3.48 and 1.47 are used for Si and SiO_2_ respectively. The variation of the resonance angle for both TE and TM polarized light with different thicknesses of Si are shown in Figure 6(a) for both water and air above the surface. Resonances with FWHM around 0.003° are calculated, due to the zero loss assumed for the layer materials. The mode profile for the first TE mode is shown in Figure 6(b). This mode is calculated for 40 nm thick silicon layer with air above the surface. This mode is similar to the above-discussed TE-polarized mode for the five-layer TiO_2_/SiO_2_ structure. The field distribution for the first TM polarized mode calculated for the 170 nm thick silicon layer with air above the surface is shown in Figure 6(c). The mode is an evanescent mode with an effective refractive index of 1.524. The sensitivity of the resonance was calculated by changing the overlying medium with a 133 nm thick silicon layer. This silicon thickness of 133 nm was selected to get a strong resonance with water above the surface. The refractive index above the surface was changed from 1.33 (water) to 1.47 (IPA) to calculate a bulk sensitivity of 1950 nm/RIU. The calculated field distribution at resonance for water above the surface is shown in the inset of Figure 6(d). The penetration depth of the electric field into the water is 300 nm. The resonance can also be excited by grating coupling or tapered coupling techniques as are used in silicon photonics.

## 3. BSW for sensing applications

**Figure 7.**
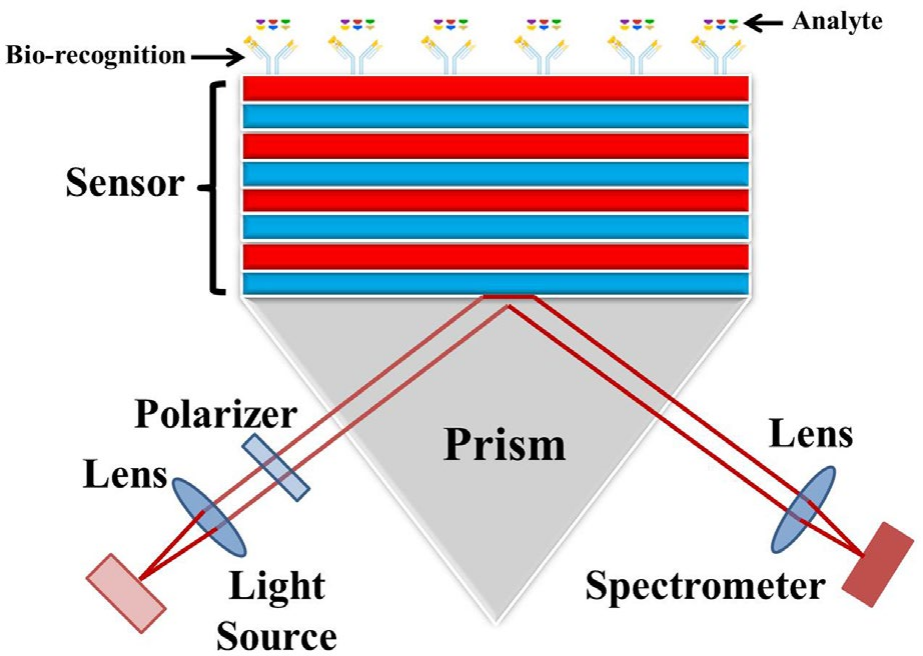
Sketch of a BSW based sensor. A bio-recognition layer is attached to the surface to make the sensor selective. Only the desired analytes can attach to the recognition layer resulting in a change in refractive index above the surface. The sensor surface can be separated into arrays of different bio-recognition analytes in order to obtain a multichannel response.

An optical sensor revolves around the transducer to inter-relate the optical and (bio) chemical domains which transforms changes in the quantity of interest into changes in the optical properties of the incident light. Typically, a bio-recognition layer is attached to the surface of the transducing medium to make the sensor selective. The interaction of the target analyte with the recognition layer produces a change in the refractive index at the surface as shown schematically in Figure 7. The sensitivity of the sensors is defined as the derivative of the monitored optical parameter (resonance angle, wavelength, intensity or phase) with respect to the parameter to be determined (e.g. refractive index and concentration). The resolution or limit of detection (LoD) is the minimum change in the parameter to be determined which can be resolved by a sensing device. The influence of the light source, photo-detector resolution and sensitivity along with temperature variations play important roles in determining the sensor resolution and cost. BSW based sensing using different materials, number of layers and sensing techniques (fluorescent and label-free) have been reported. Measurement techniques have used angular, spectral, intensity and phase interrogation. We list some of the reported sensors in descending order of the used number of layers.

Size-selective sensing of molecules in a label-free manner was demonstrated using a 21 layer thick porous-silicon based sensor.[35,36] The BSSW described earlier, confined inside but near the top of the structure, was used to sensitively detect small molecules (0.8 nm) that could infiltrate the surface. The BSW being confined at the surface was used to sense larger (60 nm) immobilized analytes. The small molecules infiltrated the top surface of the sensor and resulted in shifting of the sub-surface modes. A BSSW sensitivity of 72°/RIU (refractive index unit) or 2038 nm/RIU was reported. A BSW sensitivity of 31°/RIU or 967 nm/RIU was reported for the large molecules.

An intensity interrogation was used to improve the performance of a 20 layer TiO_2_/SiO_2_ BSW sensor.[24] An angular sensitivity of 41°/RIU was demonstrated using different concentrations of glycerol above the sensor surface. This reported sensitivity was comparable to SPR sensors.[8] The authors fixed a resonant angle and measured the change in intensity at that angle of incidence for different concentrations of glycerol above the surface. These measurements for the same sensor demonstrated better intensity sensitivities than SPR sensors due to the sharpness of the BSW resonance. They reported an intensity sensitivity of 156/RIU [45] with a limit of detection as low as 7.5 × 10^−7^ RIU, significantly better than the gold SPR sensors having a sensitivity of 24/RIU.[27]

A sensing platform capable of working simultaneously in a label-free and in a fluorescence mode was reported using a nine-layer Ta_2_O_5_ and SiO_2_ based structure.[39] Concerning the label-free operation, the reflectance was measured above the total internal reflection edge for known concentrations of glucose in doubly ionized water to show an angular sensitivity of *S*_BSW_ = 14.7°/RIU. The structure was designed to sustain a TE polarized resonance around 804 nm. The reported sensitivity was much smaller than the reported SPR and other BSW sensitivities. However, a figure of merit (FoM) was defined as:

$$\text{FoM}=S*\frac{D}{W}$$

where S is the sensitivity, D is the depth of the resonance and W represents the width of the resonance dip. The sensor was demonstrated to have a FoM_BSW_ = 82/RIU, better than the SPR sensors having FoM_SPR_ = 48/RIU. In the fluorescence mode, the surface of the sensor was labelled with dye molecules and excited by an external laser beam. The emission is dependent on the refractive index of the surface layer and is preferentially emitted into the substrate where it can be collected. Luminescence/fluorescence detection was also demonstrated using an eight-layer Ta_2_O_5_/SiO_2_ structure designed for a TE polarized resonance in the visible range.[26] The enhanced luminescence emission from the last SiO_2_ layer terminating the reflector was due to the near-field enhancement. The measurement was defined as label-free since the luminescence was concerned with the photonic structure itself rather than a specifically labelled analyte. The reported sensitivity for this sensor was 2500 nm/RIU with a limit of detection as low as 3 × 10^−6^ RIU. The Ta_2_O_5_/SiO_2_ structures used for luminescence/fluorescence detection were functionalized with bio-recognition layers and were used for detection of tumours.[46,47]

Phase detection was used to enhance the performance of a five-layer TiO_2_ and SiO_2_ based sensor because the phase of the reflected light from the BSW sensor changes rapidly as the excitation of BSW occurs.[25] Variable angle spectroscopic ellipsometry was used for both the angular interrogation and phase detection. Different concentrations of NaCl in water were used to demonstrate an angular sensitivity of 40°/RIU with a LoD of about 1.2 × 10^−4^ RIU. The calculated phase sensitive sensitivity of the sensor was 6.6 × 10^3^ °/RIU, higher than the angular sensitivity.

**Figure 8.**
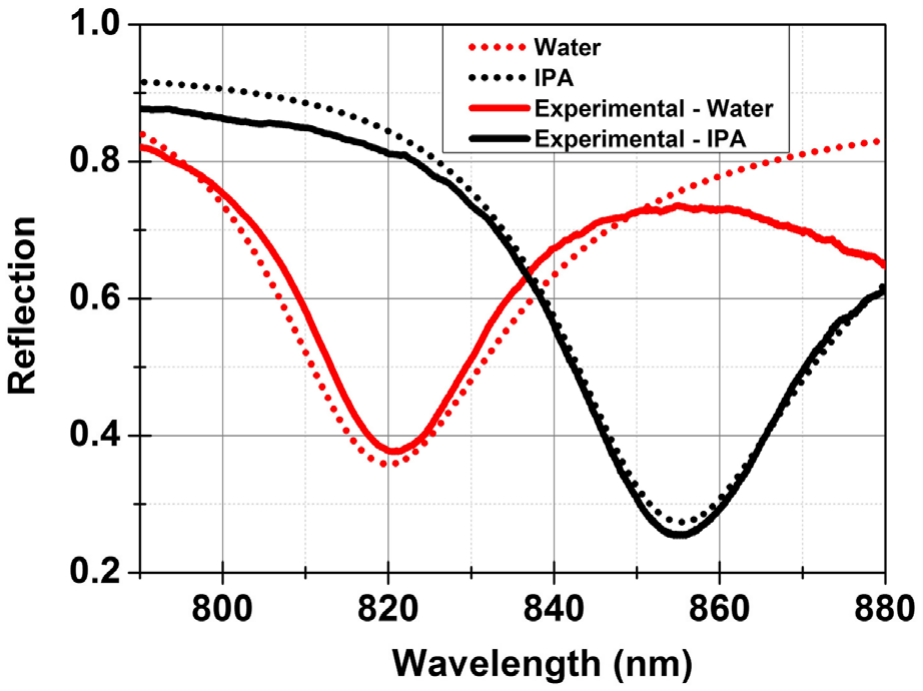
Calculated (continuous lines) and experimentally measured (dotted lines) reflectance spectra for a high index contrast sensor with water and IPA environments. The measurement is in the K-R configuration with the light incident through a 45° dove shaped sapphire prism with Durimide 112A as an index matching polymer.

A dual layer high index contrast sensor using Si and SiO_2_ with thickness of 70 nm and 676 nm deposited on sapphire was investigated. A white light source was collimated and polarized in the desired TM orientation while the reflected light was coupled to a fibre using a lens and detected by a spectrometer. A microfluidic delivery system was mounted on the sensor. A resonance at 820 nm was measured with a FWHM ~ 23 nm. Solutions having different refractive index values were prepared by mixing different percentages of IPA in water. These solutions were injected into the microfluidic channel and the resonance shift measured. The resonance was found to shift to larger wavelength values with increasing concentration of IPA in water. The relation between the increase in concentration and shift in the resonance wavelength was found to be linear. A resonance shift of Δ*λ* = 36 nm was measured for a refractive index change of Δ*n* = 0.04, i.e. changing the fluid from pure water having an index of 1.33 to pure IPA having an index of 1.37. A good agreement was found between the experimentally measured and calculated resonances as shown in Figure 8. The wavelength sensitivity of the sensor was experimentally found to be 900 nm/RIU.

Table 1 below compares the attributes of a selection of reported BSW sensors.

**Table 1.** A comparison between BSW based sensors.

No. of layers	Material (H/L)	Operating wavelength	Sensitivity		Reference
21	Porous silicon	1550 nm	BSSW	72°/RIU or 2038 nm/RIU	[36]
			BSW	31°/RIU or 967 nm/RIU	
20	Silicon nitride	1550 nm	1100 nm/RIU		[48]
20	TiO_2_/SiO_2_	980 nm	Resonance shift	41°/RIU	[24]
			Intensity interrogation	BSW - 156/RIU SPR - 24/RIU	
9	Ta_2_O_5_/SiO_2_	804 nm	14.7 °/RIU		[39]
9	Si_3_N_4_/SiO_2_	632 nm	40°/RIU		[30]
8	TiO_2_/SiO_2_ stack with a 6 nm Si layer on top	632 nm	>1000 nm/RIU		[22]
8	Ta_2_O_5_/SiO_2_	543 nm	Theoretical = 40°/RIU		[29]
			Experimental = 18°/RIU		
8	TiO_2_/SiO_2_	467 nm	600 nm/RIU		[23]
8	Ta_2_O_5_/SiO_2_ fluorescent protein on top of the stack	532 nm	2500 nm/RIU		[26]
5	TiO_2_/SiO_2_	550 nm	Reflection	40 °/RIU	[25]
			phase	6000 °/RIU	
2	Si/SiO_2_	820 nm	900 nm/RIU		

**Figure 9.**
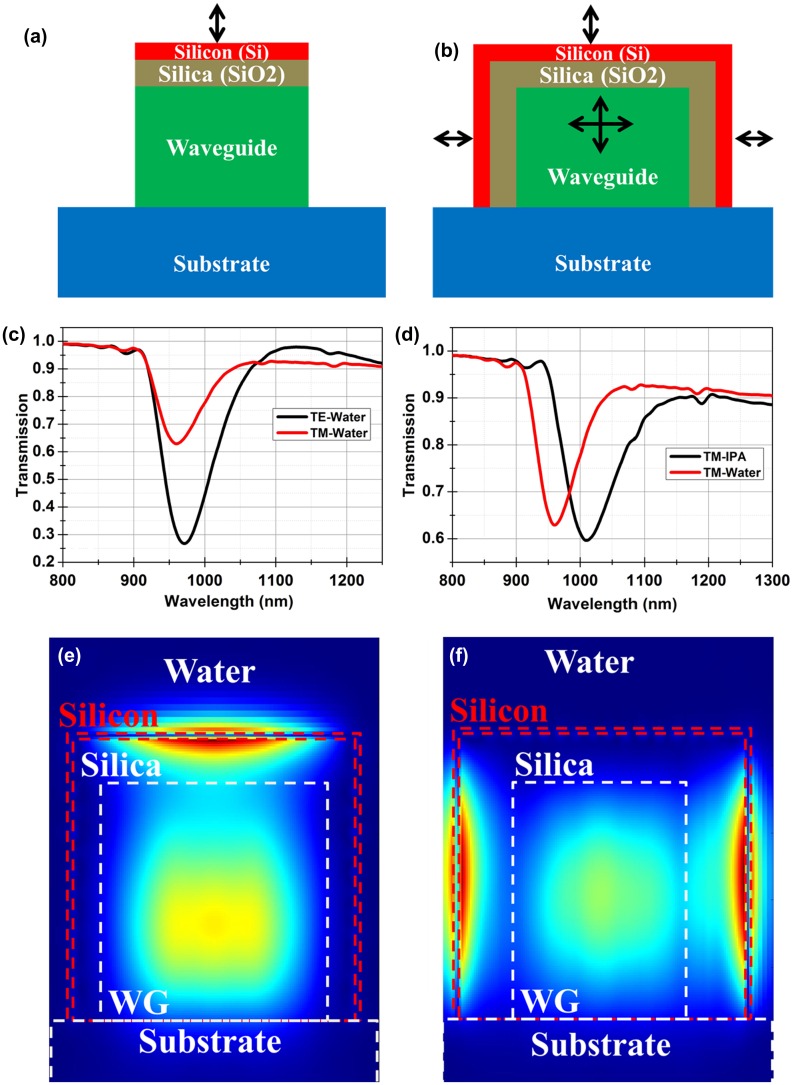
(a) Schematic of proposed waveguide (WG) coupled structures using (a) deposition of the resonant structure on the top surface of the waveguide only. The resonance can only be excited by TM polarized light. (b) Deposition of the resonant structure on three sides of the waveguide. The resonances can be excited for both TE and TM polarized light. The orientations of the electric field responsible for excitation of the resonance are marked. (c) Calculated transmission for TE and TM polarized light. These responses were calculated for a waveguide covered by sensor layers on all sides. Calculations show that TE resonance is stronger as it is excited on two sides of the waveguide. (d) Calculated shift in resonance for water (*n* = 1.33) and IPA (*n* = 1.37) environments. (e) The calculated electric field distribution for TM polarized light at resonance shows that surface wave is excited on the top surface only. (f) The calculated electric field for TE polarized light at resonance shows that surface wave is excited on both sides of the waveguide.

A new approach based on a waveguide coupled BSW resonance is proposed here as a means to avoid the use of the prism used in the K-R configuration. Dual layer Si/SiO_2_ with thicknesses of 70 and 676 nm (as discussed earlier) are to be deposited on a deep etched waveguide which will be excited by end fire coupling for excitation of BSW-like resonance at 820 nm wavelength. The dielectric layers can either be deposited only on the top surface or on all three sides of the waveguide as shown in Figure 9(a) and (b). The orientation of the electric field (polarization) responsible for the excitation of the surface wave is indicated. Only TM polarized light, having its electric field perpendicular to the deposited layers, can excite the surface wave for the structure shown in Figure 9(a). On the other hand, both TE and TM polarized light can excite the surface waves on the structure shown in Figure 9(b) where the layers are also deposited on the sides of the waveguide. The BSW will only be excited if the effective index of the waveguide mode matches.

Simulations were performed to demonstrate the working of this waveguide coupled structure. A polymer with a refractive index of 1.52 was chosen as a waveguide material with a SiO_2_ substrate. The refractive index of the polymer should be larger than SiO_2_ to prevent coupling of the light to the substrate. A 3.5 μm × 3.5 μm waveguide will achieve a waveguide mode with effective index of 1.54 (i.e. to match the effective index of the BSW). The calculated transmission responses for the structure shown in Figure 9(b) are shown in Figure 9(c) for orthogonal polarizations. Water was used as the overlying medium for these calculations. It can be noticed that the resonance dip for TM is shallower and sharper than for the TE polarized light. The resonance for TE is deeper due to the presence of two resonances. The loss associated with the material plays an important role in the sharpness of the resonance. The loss for the TE polarized resonance is larger than for the TM, resulting in broadening of the TE resonance as there are two surface waves travelling on the silicon surface. The field distributions at resonance for TM and TE polarized light are shown in Figure 9(e) and (f), confirming that the extra depth and width for the TE mode is due to the resonances on both sides of the waveguide. The shift in the resonance wavelength for a change in the refractive index of Δ*n* = 0.04 in the overlying medium, as appropriate to isopropyl alcohol (IPA), was calculated to be Δ*λ* = 50 nm (Figure 9(d)). The calculated bulk sensitivity for the TM polarized resonance is 1275 nm/RIU.

## 4. BSW for planar optical circuits

BSW, having smaller losses than SPR, are good candidates for planar optical integration and can be used as a platform for 2D planar optics in a similar manner to the use of surface plasmonic circuits. Light can be guided, focused and reflected to create an integrated optical circuit with low profile structuring. As shown already, very sensitive and localized detection can be arranged due to the strong mode confinement at the surface. Active functions can be integrated by using silicon as the surface layer through carrier injection and thermal control. An integrated Ge detector can also be envisaged. The propagation length achievable for the BSW is associated with residual material absorption, scattering and the radiation loss. The latter is reduced with a high quality factor (Q) mode. Adding a laterally structured overlayer to act as the waveguide results in a shift of effective index of the mode and the BSW resonance. The layer can be shaped by using optical lithography, stamping or other replication techniques. Guiding is obtained by changing the thickness or width of the surface layer as there is a strong dispersion in effective index with these parameters.

**Figure 10.**
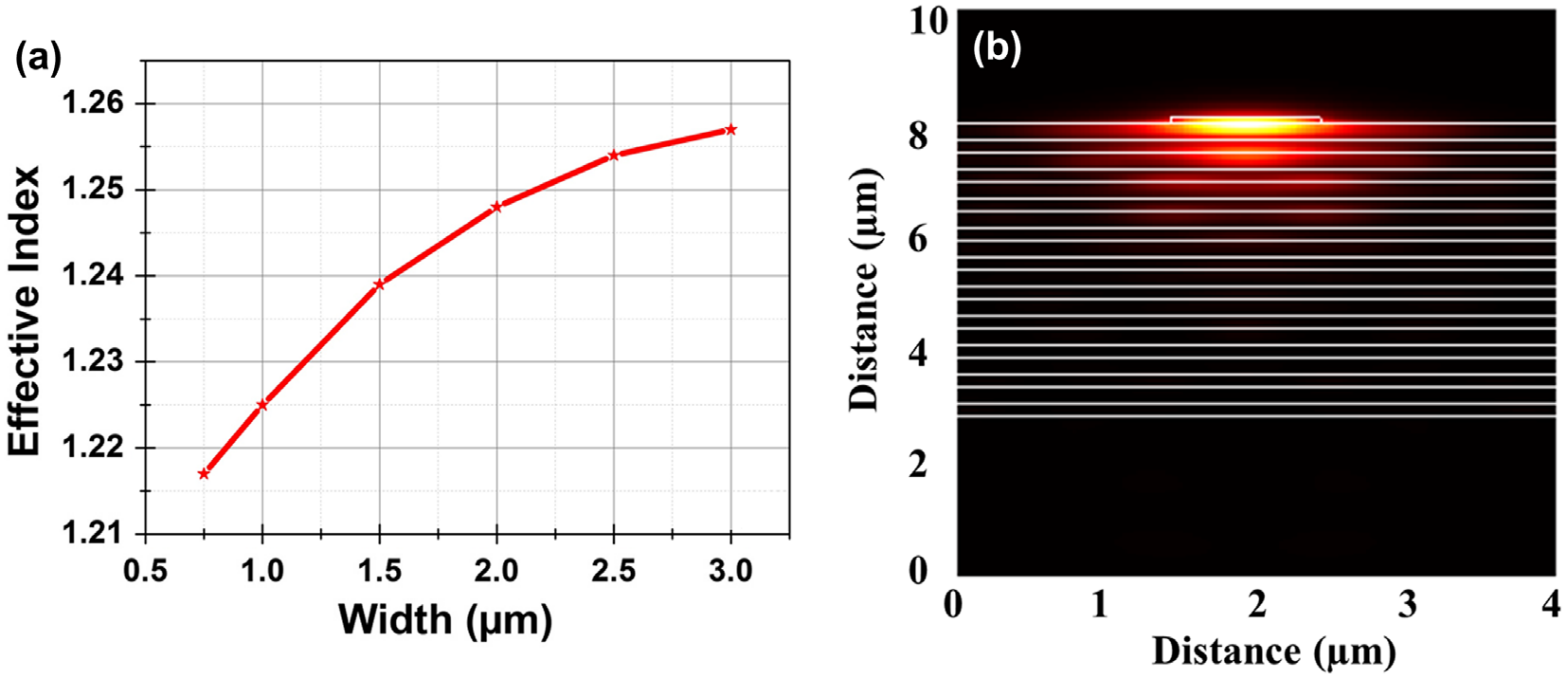
(a) Calculated effective indices of the BSW mode for different widths of the 110 nm thick polymer ridge waveguide. The effective index increases with an increase in width because the field is being concentrated in the higher index polymer. (b) Calculated mode for a 1 μm wide waveguide.

As an example, ultrathin polymeric ridges have been implemented to obtain lateral confinement of the mode in a surface waveguide [49] using a thin (110 nm) polymeric ridge of index 1.625 on top of a 20 layer stack of SiN with controlled refractive indices (*n*_H_ = 2.23, *n*_L_ = 1.75) at telecom (1530 nm) wavelengths where tuneable lasers are readily available. The polymer ridge supports a laterally guided TE-polarized BSW. The effective index depends strongly on the thickness of the polymer. Calculations show a change in effective index of 0.012 for a 20 nm change in thickness around the baseline (110 nm) which implies that high optical confinement can be obtained enabling small bend radii. The effective index increases with increasing width of the waveguide. The effective indices and calculated mode for ridge width of 1 μm are shown in Figure 10.

**Figure 11.**
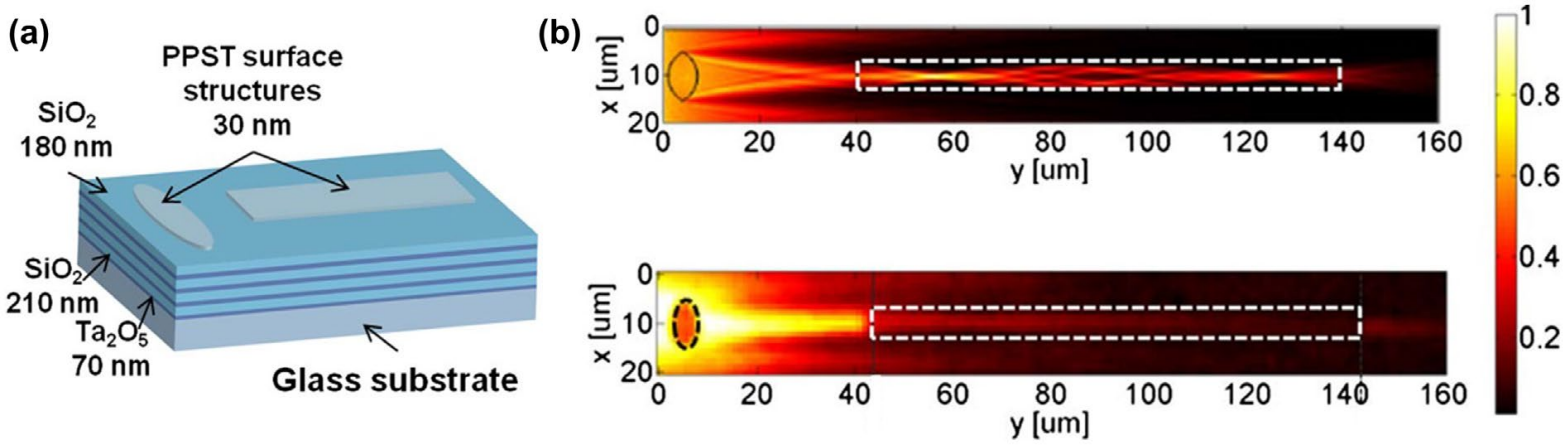
(a) SiO_2_/Ta_2_O_5_ multilayer with plasma-deposited polystyrene (PPST) refractive structures on the surface. (b) Calculated (top) and measured (bottom) intensity distribution of a BSW injected into a polymeric ridge waveguide (contour, dashed white line) by means of a planar lens. Reproduced with permission from [50].

The guiding of BSW in lateral waveguides has been directly measured by using scanning near-field optical microscopy (SNOM) [40,49] and by fluorescence microscopy with luminescent molecules attached to the surface.[31] The manipulation of BSW [33,42] on a surface has been demonstrated. For example, a planar lens was implemented using a shaped polymer layer on a 12-layer dielectric multilayer operating at a wavelength of 1500 nm. Focusing and injection into a thin waveguide was studied and it was shown that the propagation length is dependent on the Q of the resonance.[50] A BSW propagation length of 2.5 mm was recently demonstrated using a TiO_2_ waveguiding layer.[51]

## 5. Summary and outlook

We have introduced the resonant properties of BSW modes and the opportunity to engineer the electromagnetic field at the surface of a dielectric reflector. A desired wavelength, angle, FWHM and polarization for the resonance can be obtained through careful design and does not require lateral structuring. These BSW resonances can be used as the basis of label-free sensors providing larger sensitivities and lower detection limits than metal based SPR sensors. The high field enhancement can be used to selectively excite fluorescence in labelled molecules attached to the surface. A waveguide coupled BSW suitable for on-chip sensing applications using end-fire coupling is proposed which we believe will define a significant role for BSW in sensing technologies. The high index contrast of the SOI system allows to design sensors on the emerging silicon photonics platform operating at telecom wavelengths. Guiding and manipulation of the BSW is also discussed to give an insight into BSW applications other than sensing. Despite their discovery many years ago, BSW are at a relatively early stage in their development and new opportunities and applications are likely. These dielectric based surface waves will complement that of metal based surface plasmons.

## Disclosure statement

No potential conflict of interest was reported by the authors.

## Funding

This work is funded by Science Foundation Ireland [under 12/RC/2276 (IPIC)]; and European Union’s Seventh Framework Programme [FP7/2007-2013 under grant agreement number FP7-IRSES-295182].
